# Prenatal opioid-exposed infant extracellular miRNA signature obtained at birth predicts severity of neonatal opioid withdrawal syndrome

**DOI:** 10.1038/s41598-022-09793-7

**Published:** 2022-04-08

**Authors:** Amanda H. Mahnke, Melissa H. Roberts, Lawrence Leeman, Xingya Ma, Ludmila N. Bakhireva, Rajesh C. Miranda

**Affiliations:** 1 Department of Neuroscience and Experimental Therapeutics, College of Medicine, Texas A&M University Health Science Center, 8447 Riverside Parkway, Bryan, TX 77807-3260 USA; 2 Department of Pharmacy Practice and Administrative Sciences, Substance Use Research and Education (SURE) Center, University of New Mexico College of Pharmacy, Albuquerque, NM 87131 USA; 3 Department of Family and Community Medicine, University of New Mexico School of Medicine, Albuquerque, NM 87106 USA; 4 Department of Obstetrics and Gynecology, University of New Mexico School of Medicine, Albuquerque, NM 87106 USA; 5 Division of Epidemiology, Biostatistics and Preventive Medicine, Department of Internal Medicine, University of New Mexico School of Medicine, Albuquerque, NM 87106 USA

**Keywords:** miRNAs, Predictive markers

## Abstract

Prenatal opioid exposure (POE) is commonly associated with neonatal opioid withdrawal syndrome (NOWS), which is characterized by a broad variability in symptoms and severity. Currently there are no diagnostic tools to reliably predict which infants will develop severe NOWS, while risk stratification would allow for proactive decisions about appropriate clinical monitoring and interventions. The aim of this prospective cohort study was to assess if extracellular microRNAs (miRNAs) in umbilical cord plasma of infants with POE could predict NOWS severity. Participants (n = 58) consisted of pregnant women receiving medications for opioid use disorder and their infants. NOWS severity was operationalized as the need for pharmacologic treatment and prolonged hospitalization (≥ 14 days). Cord blood miRNAs were assessed using semi-quantitative qRT-PCR arrays. Receiver operating characteristic curves and area under the curve (AUC) were estimated. The expression of three miRNAs (miR-128-3p, miR-30c-5p, miR-421) predicted need for pharmacologic treatment (AUC: 0.85) and prolonged hospitalization (AUC: 0.90). Predictive validity improved after two miRNAs (let-7d-5p, miR-584-5p) were added to the need for pharmacologic treatment model (AUC: 0.94) and another two miRNAs (let-7b-5p, miR-10-5p) to the prolonged hospitalization model (AUC: 0.99). Infant cord blood extracellular miRNAs can proactively identify opioid-exposed neonates at high-risk for developing severe NOWS.

## Introduction

Increased rates of opioid use and misuse in the general population, often termed the ‘opioid epidemic’, are reflected in the increased rates of opioid use in pregnant women. In the US, an analysis of hospital discharge data following in-hospital delivery found that among women who had just given birth, the rates of opioid use disorder (OUD) increased more than threefold from 1999 to 2014^1^. During pregnancy, medication for opioid use disorder (MOUD), consisting of either the partial opioid receptor agonist, buprenorphine, or the full opioid receptor agonist, methadone, is the recommended treatment modality for OUD^2,3^. Both chronic opioid use and MOUD can result in neonatal opioid withdrawal syndrome (NOWS), formerly/alternatively labeled as neonatal abstinence syndrome (NAS). Rates of NOWS have also drastically risen, increasing more than 1.5-fold from 2009 to 2017^4^.

NOWS symptoms can include respiratory, gastrointestinal, and feeding problems and often require prolonged hospitalization. The long-term consequences of prenatal opioid exposure (POE) are unclear as it is often hard to separate the effect of POE from contributing effects of other substances and adverse pregnancy and perinatal environments. Published reports indicate that POE is associated with infant emotional regulation and stress reactivity^5,6^, systemic inflammation^7,8^, and altered neurodevelopment^9–11^, however, other studies have been generally reassuring with respect to the effects on cognitive development^6,12,13^. It is estimated that 31–97% of neonates with POE will develop at least some withdrawal symptoms^14–16^, though there is substantial variability in the incidence, onset, duration, and severity of NOWS. No single factor, or combination of known factors, has sufficiently explained the variability in NOWS severity^17,18^. While some protective factors have been identified, i.e. the type of maternal MOUD (buprenorphine vs. methadone), initiation of breastfeeding, rooming-in practices, non-pharmacological interventions, and genetics^18–22^, large unexplained variability in the severity of NOWS symptoms suggest that additional factors might be at play. Current tools to measure NOWS severity rely on the detection and scoring of symptoms^23–28^. One predictive tool recently created is based on maternal and infant characteristics to indicate risk of NOWS development^29^, but this tool does not capture underlying biological vulnerabilities within the neonate, such as epigenetic factors. Consequently, there is an unmet need for biomarkers to proactively identify neonates at high-risk and low-risk for NOWS development to improve observation, treatment, and outcomes for infants with POE.

MicroRNAs (miRNAs) are small non-coding RNA molecules that intracellularly regulate RNA translation^30,31^, but can also be released extracellularly, including into the circulation, where they are thought to be a mechanism of cell-to-cell communication^32^. Extracellular miRNA profiles are altered by opioid exposure. A study in healthy adult male volunteers found 96 plasma circulating miRNAs altered by 24 h of either hydromorphone or oxycodone exposure, with 27% of these miRNAs altered similarly by both drug exposures^33^. Extracellular serum miRNAs were found to differentiate alcohol- and opioid-using pregnant women from alcohol- and opioid-naïve pregnant women^34^. Circulating miRNAs have also been shown to predict outcomes following prenatal exposures and, potentially, mediate of the effects of those prenatal exposures. We previously found that plasma miRNAs assessed at mid-pregnancy could predict future adverse birth outcomes following alcohol exposure, i.e., identify infants with neurobehavioral and growth deficits associated with fetal alcohol spectrum disorders from unaffected infants^35,36^. These maternal miRNAs that were predictive of infant outcomes were shown, in preclinical models, to produce placental and fetal growth deficits, recapitulating, in the absence of alcohol, the intrauterine growth restriction seen with prenatal alcohol exposure^37^. Recently, we have shown that plasma miRNAs in early infancy can also be predictive of growth deficits and neurobehavioral outcomes following prenatal alcohol exposure^38^. These studies show that extracellular miRNA expression is altered by prenatal drug exposures and can be indicative of future adverse health outcomes.

The primary objective of the current study was to determine if extracellular plasma miRNAs, derived from the umbilical cord at birth, could predict the severity of NOWS, as determined by the need for pharmacologic treatment with opioids and length of hospital stay. We hypothesized, given our previous research showing that circulating miRNAs could predict infant outcomes following ethanol exposure, that neonatal circulating miRNAs would have predictive value for both metrics of NOWS severity.

## Results

### Participant characteristics

Participant demographic characteristics are summarized in Table 1. Mean maternal age at recruitment was 28.7 ± 5.8 years and participants were recruited, on average, during the second trimester (mean gestational age at recruitment: 21.5 ± 7.2 weeks). The sample was racially and ethnically diverse (74.1% Latinx and 6.9% Native Americans). Approximately a third of the women (34.5%) reported less than high school education level. Maternal substance use is summarized in Table 2. Alongside buprenorphine and methadone MOUD, concurrent use of other opioids, i.e., heroin (36.2%) or misuse of opioid analgesics (37.9%), was prevalent but similar in both groups, and no group differences in use of alcohol, tobacco, marijuana, benzodiazepines or other substances were observed. Participants in both groups were positive for one alcohol biomarker at delivery (*Not-Pharmacologically-Treated* Maternal: gamma-glutamyl transpeptidase [GGT] 4.9%, carbohydrate deficient transferrin [%dCDT] 2.4%,urine ethyl sulfate [uEtS] 9.8%; Infant: phosphatidylethanol [PEth] 12.2%; *Pharmacologically-Treated* Maternal: GGT 5.9%, uEtS 11.8%; Infant: PEth 23.5%) but the rates of positivity for a single alcohol biomarker at delivery and for each biomarker type were not different between groups. A substantially higher proportion of *Pharmacologically-Treated* infants (64.7%) were prenatally exposed to methadone compared to *Not-Pharmacologically-Treated* (24.4%; p < 0.01). There were no differences in maternal characteristics between the study groups except for a higher prevalence of Hepatitis C in *Pharmacologically-Treated* group (35.3%) compared to *Not-Pharmacologically-Treated* (22.0%) group (p = 0.03).

**Table 1:** Maternal socio-demographic and medical characteristics.

Variable	Not-Pharmacologically-Treated (N = 41)	Pharmacologically-Treated (N = 17)	p-value
	Mean ± SD	Mean ± SD	
Maternal age at enrollment (years)	29.2 ± 5.9	27.2 ± 5.4	0.23°
Gestational age at enrollment (weeks)	22.1 ± 7.4	20.2 ± 6.8	0.41^1^
Body mass index (BMI)	25.2 ± 5.2	24.6 ± 5.9	0.68°

	N (%)	N (%)	
**Race**			0.79^3^
White	37 (90.2%)	15 (88.2%)	
American Indian	3 (7.3%)	1 (5.9%)	
Other or Mixed	1 (2.4%)	1 (5.9%)	
Ethnicity: Hispanic/Latina	31 (75.6%)	12 (70.6%)	0.75^3^
**Marital/cohabiting status**			0.26^3^
Single/separated/divorced	19 (46.3%)	5 (29.4%)	
Married/cohabiting	22 (53.7%)	12 (70.6%)	
**Educational level**			0.69^2^
Less than high school graduate	15 (36.6%)	5 (29.4%)	
High school graduate or GED	10 (24.4%)	6 (35.3%)	
At least some college or vocational school	16 (39.0%)	6 (35.3%)	
Employed (at enrollment)	17 (41.5%)	5 (29.4%)	0.55^3^
**Health insurance**			0.35^3^
Employer-based insurance/self-purchased/other	2 (4.9%)	1 (5.9%)	
Medicaid	39 (95.1%)	16 (94.1%)	
Gravidity: primigravida	5 (12.2%)	4 (23.5%)	0.43^3^
Parity: nulliparous	8 (19.5%)	6 (35.3%)	0.20^2^
**Medical conditions**			
Hepatitis C	9 (22.0%)	6 (35.3%)	0.03^2^
Preeclampsia or hypertension	6 (14.6%)	5 (29.4%)	0.19^2^

*GED* graduate equivalency degree, *SD* standard deviation.

°Based on pooled variances t-test.

^1^Based on Mann–Whitney test.

^2^Based on Chi-square test.

^3^Based on Fisher's exact test.

**Table 2:** Maternal substance use patterns.

Variable	Not-Pharmacologically-Treated (N = 41)	Pharmacologically-Treated (N = 17)	p-value
	N (%)	N (%)	
**MOUD**			< 0.01^3^
Buprenorphine	31 (75.6%)	6 (35.3%)	
Methadone	10 (24.4%)	11 (64.7%)	
**Stimulant Use at Visit 1**^**4**^	6 (14.6%)	1 (5.9%)	0.66^3^
Methamphetamines	6 (14.6%)	1 (5.9%)	0.66^3^
Cocaine	1 (2.4%)	0 (0.0%)	1.00^3^
**Substance use across Visit 1 and Visit 2**^**5**^			
Other opioid use			
Heroin	15 (36.6%)	6 (35.3%)	0.93^2^
Misuse of opioid analgesics	16 (39.0%)	6 (35.3%)	0.79^2^
Benzodiazepines	12 (29.3%)	3 (17.6%)	0.36^2^
Sedatives	2 (4.9%)	1 (5.9%)	1.00^3^
Marijuana	18 (43.9%)	7 (41.2%)	0.85^2^
Other substances	2 (4.9%)	0 (0.0%)	1.00^3^
Tobacco use	36 (87.8%)	16 (94.1%)	0.47^2^
Positive for 1 ethanol biomarker at delivery	12 (29.3%)	7 (41.2%)	0.25^2^

	Mean ± SD	Mean ± SD	
Absolute alcohol(oz) per day, TLFB around LMP	0.80 ± 2.2	0.03 ± 0.1	0.07^1^
Absolute alcohol(oz) per day, TLFB Visit 1	0.00 ± 0.01	0.00 ± 0.00	0.54^1^
Absolute alcohol(oz) per day^6^	0.40 ± 1.10	0.02 ± 0.06	0.06^1^

*LMP* last menstrual period, *MOUD* medications for opioid use disorder, *NOWS* neonatal opioid withdrawal syndrome, *SD* standard deviation, *TLFB* timeline follow-back.

^1^Based on Mann–Whitney test.

^2^Based on Chi-square test.

^3^Based on Fisher's exact test.

^4^Any use of methamphetamines, cocaine, crack, MDMA, PCP, or other inhalants (more than occasional use of stimulants anytime in pregnancy and any use after the first trimester) were exclusionary criteria; stimulants not shown had no evidence of use.

^5^Visit 1 occurred prenatally and Visit 2 occurred at delivery/birth.

^6^Average use from 2 TLFB calendars (around LMP and Visit 1).

Perinatal and infant characteristics are summarized in Table 3. In the study population of 58 infants, 17 received pharmacological treatment, and 15 had a hospital stay ≥ 14 days. There were differences in the mean gestational age at delivery (36.9 ± 1.0 vs. 38.8 ± 1.4 weeks; p < 0.0001) reflected in the higher incidence of preterm delivery in the *Pharmacologically-Treated* group compared to *Not-Pharmacologically-Treated* group (23.5% vs. 4.9%; p = 0.06). Length of hospital stay (LOS) was significantly longer in the *Pharmacologically-Treated* (19.5 ± 9.5 days) compared to *Not-Pharmacologically-Treated* group (6.0 ± 3.6 days; p < 0.01). Respiratory distress was also significantly more prevalent in the *Pharmacologically-Treated* compared to *Not-Pharmacologically-Treated* group (47.1% vs 9.8%, p < 0.01). Type of maternal MOUD exposure was buprenorphine for 37 (63.8%) infants and methadone for 21 (36.2%) infants. Higher percentages of infants with methadone exposure experienced the two main outcomes, pharmacological treatment and LOS ≥ 14 days. There was substantial overlap between these outcomes; of the 17 infants receiving pharmacological treatment, 13 (76.5%) had a LOS ≥ 14 days. Of the 6 infants with maternal buprenorphine exposure, 3 (50%) had a LOS ≥ 14 days, while among the 11 infants with maternal methadone exposure, 10 (90.9%) had a LOS ≥ 14 days. No other differences in infant characteristics were observed.

**Table 3:** Perinatal and infant characteristics.

Variable	Not-Pharmacologically-Treated (N = 41)	Pharmacologically-Treated (N = 17)	p-value
	Mean ± SD	Mean ± SD	
Gestational age at delivery (weeks)	38.8 ± 1.4	36.9 ± 1.0	< 0.001^1^
Infant birth weight (grams)	2999.0 ± 453.6	2702.7 ± 249.7	0.01°
Length of hospitalization (days)	6.0 ± 3.6	19.5 ± 9.5	< 0.001^1^
APGAR score at 1 min	8.0 ± 0.6	7.5 ± 1.5	0.10^1^
APGAR score at 5 min	8.7 ± 1.4	8.7 ± 0.8	0.41^1^

	N (%)	N (%)	
Preterm delivery (< 37 weeks)	2 (4.9%)	4 (23.5%)	0.06^3^
Delivery type: vaginal	35 (85.4%)	12 (70.6%)	0.19^2^
APGAR score < 7 at 1 min	1 (2.4%)	1 (5.9%)	0.50^3^
APGAR score < 7 at 5 min	1 (2.4%)	1 (5.9%)	0.50^3^
Infant’s gender: boy	21 (51.2%)	7 (41.2%)	0.49^2^
Length of hospitalization ≥ 14 days	2 (4.9%)	13 (76.5%)	< 0.001^3^
**Neonatal complications**			
Hyperbilirubinemia requiring phototherapy	16 (39.0%)	8 (47.1%)	0.77^3^
Respiratory distress	4 (9.8%)	8 (47.1%)	< 0.01^3^
Hypoglycemia	0 (0.0%)	2 (11.8%)	0.08^3^
Tachypnea	1 (2.4%)	2 (11.8%)	0.20^3^
Bradycardia	3 (7.3%)	2 (11.8%)	0.62^3^
Sepsis	0 (0.0%)	0 (100.0%)	1.00^3^

*APGAR* appearance, pulse, grimace, activity, and respiration score, *NOWS* neonatal opioid withdrawal syndrome, *SD*, standard deviation.

°Based on pooled variances t-test.

^1^Based on Mann–Whitney test.

^2^Based on Chi-square test.

^3^Based on Fisher's exact test.

### Plasma and RNA characteristics

#### Sample purity characteristics

There were no significant differences in hemolysis measures, including the presence of free hemoglobin and difference in cycle threshold (CT) for hemolytic markers (ΔCT_hemolysis_), when comparing *Pharmacologically-Treated* to *Not-Pharmacologically-Treated* or *LOS* ≥ *14 days* to *LOS* < *14 days* (all p > 0.10). There was also no significant correlation between sample absorbance at 414 nm and ΔCT_hemolysis_ (r = 0.22, p = 0.094), therefore, samples were excluded from analysis only if it exceeded criterion on both measures^38^. There were no between-group differences in plasma total RNA concentration, qPCR amplification, or technical performance on focus panels (Supplementary Fig. S2, all p > 0.10).

### miRNA expression by NOWS severity outcomes

**Figure 1:**
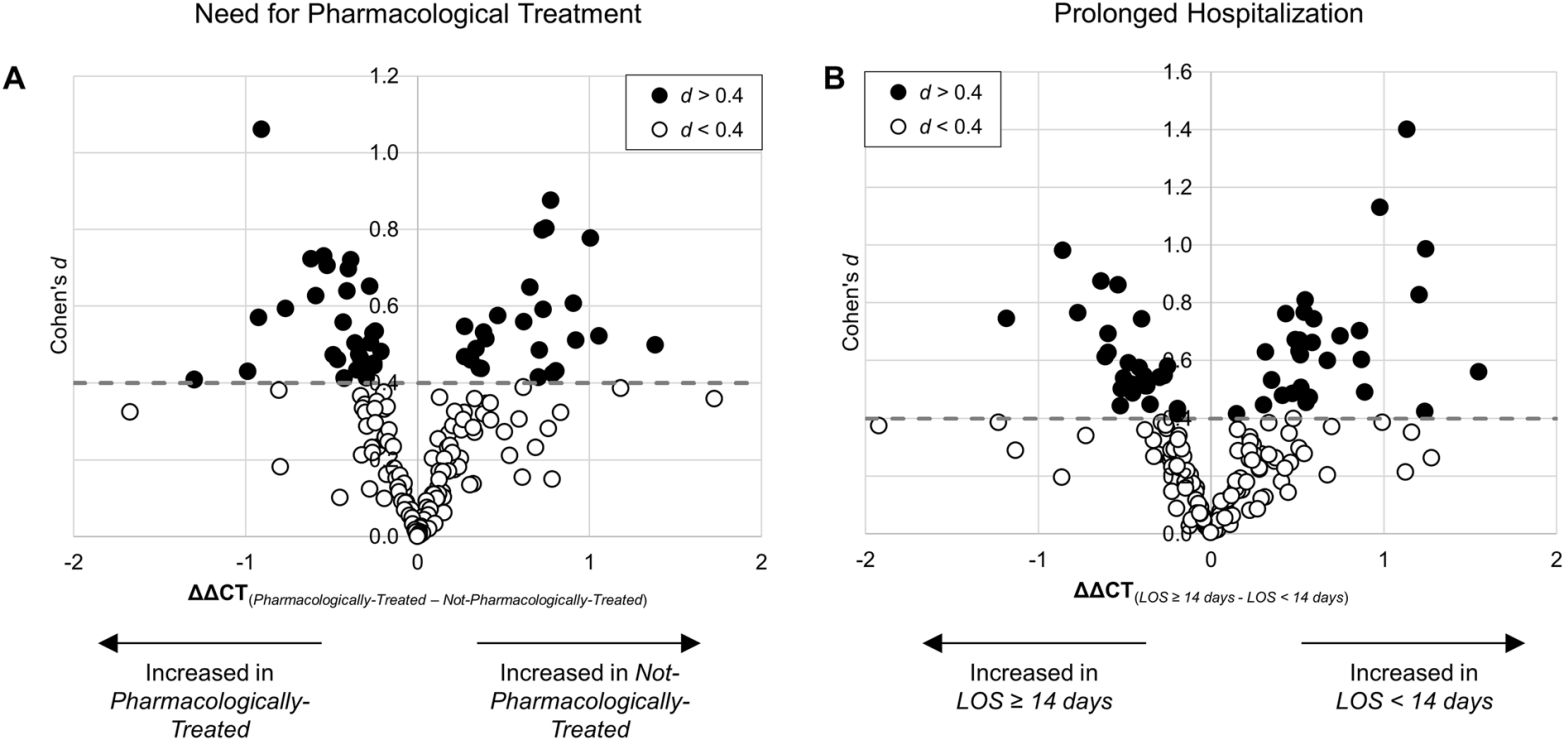
miRNA Expression in Infants with NOWS Requiring Pharmacologic Treatment or Prolonged Hospitalization. Volcano plots of cord blood plasma extracellular miRNA expression (difference in normalized CT between groups, ΔΔCT) and effect size (Cohen’s *d*) comparing miRNA expression in neonates *Pharmacologically-Treated* compared to *Not-Pharmacologically-Treated* (**A**) and in *LOS* ≥ *14 days* compared to *LOS* < *14 days* neonates (**B**). White filled points denote miRNAs altered with a small effect size (*d* < 0.4), black filled points denote miRNAs with moderate or larger effect size (*d* > 0.4), and dashed line denotes *d* = 0.4.

At birth, neonates *Pharmacologically-Treated* expressed a substantially different plasma extracellular miRNA expression profile compared to neonates with POE who were *Not-Pharmacologically-Treated* (Fig. 1A, Supplementary Table S3). Of the 171 miRNAs assessed, 31.0% were altered with a moderate effect size (*d* > 0.40, Fig. 1A). Of the miRNAs altered with a moderate effect size, 45.3% were more highly expressed in *Pharmacologically-Treated* neonates. While a moderate effect size was noted for 31.0% of miRNAs, 20.8% both exceeded criterion (p < 0.10) and had a moderate effect size. In stratified analyses, 17.3% of miRNAs in neonates with maternal methadone MOUD exposure both exceeded criterion (p < 0.10) and had a moderate effect size compared to 6.5% of miRNAs in neonates with maternal buprenorphine MOUD exposure (Supplementary Table S3). When miRNA expression was compared in neonates with prolonged hospitalization (*LOS* ≥ *14 days*) to neonates with *LOS* < *14 days* (Fig. 1B, Supplementary Table S3), 35.1% of miRNAs were altered with a moderate effect size. Of the miRNAs altered with a moderate effect size, 54.7% were more highly expressed in *LOS* ≥ *14 days* neonates compared to *LOS* < *14 days* neonates. Among the miRNAs identified by comparing hospital stay, 24.4% both exceeded criterion (p < 0.10) and had a moderate effect size, and in stratified analyses, 15.5% of miRNAs in neonates with maternal methadone MOUD exposure and 13.1% of miRNAs in neonates with maternal buprenorphine MOUD exposure exceeded criterion (p < 0.10) and had a moderate effect size (Supplementary Table S3).

### miRNA which met initial eligibility criteria for inclusion in predictive models

Differences in the effect size and corresponding statistical significance for all miRNAs are presented in the Supplementary Table S3. Eligible model candidates are listed in Table 4 and include 51 miRNAs that met eligibility criteria for at least one of the two outcomes, pharmacological treatment (n = 35) or LOS ≥ 14 days (n = 41). Among those, 25 miRNAs were predictive of both NOWS outcomes, with a greater mean effect size observed for LOS ≥ 14 days than for pharmacological treatment (0.77 ± 0.19 and 0.64 ± 0.14, respectively,* p* = 0.008). Ultimately, the final parsimonious models included a signature of three miRNAs which were predictive of both NOWS outcomes (miR-128-3p, miR-30c-5p, miR-421), plus two miRNAs which were predictive of one outcome but not another (i.e., let-7d-5p and miR-584-5p for the need of pharmacologic treatment; let-7b-5p and miR-10b-5p for the prolonged hospitalization).

**Table 4:** List of miRNAs that met initial eligibility criteria^a^ for inclusion in predictive models.

miRNA	Pharmacologic treatment		LOS ≥ 14 Days	
	Difference^1^ Mean (SD)	Cohen's *d*^2^	Difference^3^ Mean (SD)	Cohen's *d*^*2*^
**miRNAs which met initial eligibility criteria for both outcomes**				
*hsa-let-7b-5p*	− 0.77 (0.88)*	0.88	− 1.13 (0.81)*	1.40
hsa-miR-125a-5p	− 0.73 (1.24)*	0.59	− 0.86 (1.22)*	0.70
*hsa-miR-128-3p*	0.41 (0.64)*	0.64	0.54 (0.62)*	0.86
hsa-miR-154-5p	0.62 (0.85)*	0.72	0.60 (0.86)*	0.69
hsa-miR-18b-5p	0.53 (0.74)*	0.71	0.64 (0.73)*	0.87
hsa-miR-21-5p	− 0.27 (0.59)†	0.47	− 0.43 (0.57)*	0.76
hsa-miR-23a-3p	− 0.47 (0.81)*	0.58	− 0.59 (0.80)*	0.74
hsa-miR-23b-3p	− 0.40 (0.77)†	0.52	− 0.51 (0.76)*	0.67
hsa-miR-24-3p	− 0.37 (0.85)†	0.44	− 0.52 (0.84)*	0.62
hsa-miR-27b-3p	− 0.34 (0.70)*	0.49	− 0.54 (0.67)*	0.81
hsa-miR-29a-3p	− 1.01 (1.30)*	0.78	− 1.24 (1.26)*	0.99
hsa-miR-29c-3p	− 0.62 (1.11)†	0.56	− 0.75 (1.09)*	0.68
hsa-miR-30a-5p	− 0.73 (0.91)*	0.80	− 0.98 (0.87)*	1.13
*hsa-miR-30c-5p*	− 0.28 (0.50)†	0.55	− 0.31 (0.50)*	0.63
hsa-miR-362-3p	0.92 (1.62)*	0.57	1.19 (1.59)*	0.74
hsa-miR-365a-3p	− 0.91 (1.50)*	0.61	− 1.21 (1.46)*	0.83
hsa-miR-382-5p	0.59 (0.95)†	0.63	0.60 (0.95)†	0.63
*hsa-miR-421*	0.91 (0.86)*	1.06	0.86 (0.87)*	0.98
hsa-miR-423-3p	0.26 (0.49)†	0.53	0.27 (0.49)†	0.55
hsa-miR-423-5p	0.28 (0.43)*	0.65	0.25 (0.43)†	0.58
hsa-miR-484	0.39 (0.54)*	0.72	0.40 (0.54)*	0.74
hsa-miR-495-3p	0.46 (1.01)†	0.46	0.61 (1.00)*	0.61
*hsa-miR-584-5p*	0.54 (0.75)*	0.73	0.48 (0.76)*	0.64
hsa-miR-629-5p	0.77 (1.29)†	0.59	0.77 (1.30)*	0.59
hsa-miR-652-3p	0.40 (0.58)*	0.70	0.50 (0.56)*	0.90
**miRNAs which met initial eligibility criteria for pharmacologic treatment**				
*hsa-let-7d-5p*	− 0.65 (1.01)†	0.65	− 0.52 (1.03)	0.51
hsa-miR-133b	− 0.92 (1.81)†	0.51	− 0.89 (1.81)	0.49
hsa-miR-146b-5p	− 0.75 (0.93)*	0.80	− 0.47 (0.97)	0.49
hsa-miR-152-3p	0.25 (0.46)*	0.53	0.19 (0.47)	0.42
hsa-miR-186-5p	0.36 (0.72)†	0.50	0.37 (0.73)	0.51
hsa-miR-18a-5p	0.43 (0.77)*	0.56	0.35 (0.78)	0.45
hsa-miR-223-5p	− 1.06 (2.02)†	0.52	− 0.02 (2.08)	0.01
hsa-miR-320d	0.27 (0.54)†	0.50	0.19 (0.55)	0.34
hsa-miR-324-5p	0.99 (2.30)*	0.43	0.11 (2.34)	0.05
hsa-miR-543	1.30 (3.18)*	0.41	1.23 (3.19)†	0.39
**miRNAs which met initial eligibility criteria for LOS ≥ 14 Days**				
hsa-let-7e-5p	− 0.39 (0.72)	0.53	− 0.54 (0.71)*	0.77
hsa-miR-103a-3p	0.18 (0.74)	0.25	0.41 (0.72)†	0.57
*hsa-miR-10b-5p*	− 0.25 (0.92)	0.28	− 0.59 (0.89)*	0.66
hsa-miR-125b-5p	− 0.40 (1.15)	0.35	− 0.67 (1.12)*	0.60
hsa-miR-127-3p	0.32 (0.95)	0.33	0.51 (0.94)†	0.54
hsa-miR-146a-5p	0.24 (0.83)	0.30	0.48 (0.81)†	0.59
hsa-miR-191-5p	0.08 (0.56)	0.14	0.30 (0.55)*	0.54
hsa-miR-27a-3p	− 0.36 (0.82)	0.44	− 0.51 (0.81)*	0.63
hsa-miR-328-3p	− 0.17 (0.75)	0.23	− 0.49 (0.73)†	0.67
hsa-miR-34a-5p	− 0.21 (1.23)	0.17	− 0.55 (1.21)†	0.46
hsa-miR-376c-3p	0.43 (1.04)	0.41	0.52 (1.03)†	0.50
hsa-miR-409-3p	0.49 (1.04)	0.47	0.77 (1.01)*	0.76
hsa-miR-425-3p	0.33 (0.90)	0.37	0.46 (0.89)†	0.52
hsa-miR-505-3p	0.13 (0.76)	0.18	0.38 (0.74)*	0.51
hsa-miR-532-5p	0.33 (0.71)	0.46	0.39 (0.71)†	0.55
hsa-miR-99a-5p	− 0.33 (0.92)	0.36	− 0.61 (0.89)*	0.69

Final model miRNAs were identified through the stepwise logistic regression modelling and are shown in italics.

*CT* cycle threshold, *ΔCT* normalized cycle threshold, *LOS* length of hospital stay, *SD* standard deviation.

^a^Criteria included: p < 0.10 for the differences in miRNA expression and *d* > 0.40 for mean difference in miRNA expression in unadjusted analyses.

^1^ΔΔCT [(− ΔCT_*Pharmacologically-Treated*_) − (− ΔCT_*Not-Pharmacologically-Treated*_)].

^2^Effect size shown as absolute value.

^3^ΔΔCT [(− ΔCT_*LOS* ≥ *14 days*_) − (− ΔCT_*LOS* < *14 days*_)].

*p < 0.05; †p < 0.10.

### Predictive validity of the common miRNA signature for identification of both NOWS severity outcomes

Receiver operator characteristic (ROC) curves were constructed for a model containing the three core miRNAs (miR-128-3p, miR-30c-5p, miR-421) to assess the predictive validity of these miRNAs for both NOWS outcomes. For the need for pharmacological treatment outcome, the area under the ROC curve (AUC) was 0.85, the overall accuracy of the miRNA signature was 86.2%, sensitivity was 58.8% (95% confidence interval [CI] 32.9; 81.6) and positive predictive value (PPV) was 90.9% (95% CI 58.7; 99.8). For the prolonged hospitalization outcome, AUC was 0.90, the overall accuracy of the miRNA signature was 87.9%, sensitivity was 66.7% (95% CI 38.4; 88.6) and PPV was 83.3% (95% CI 51.6; 97.9).

### Predictive validity of the miRNA signature for identification of infants with NOWS requiring pharmacologic treatment

**Figure 2:**
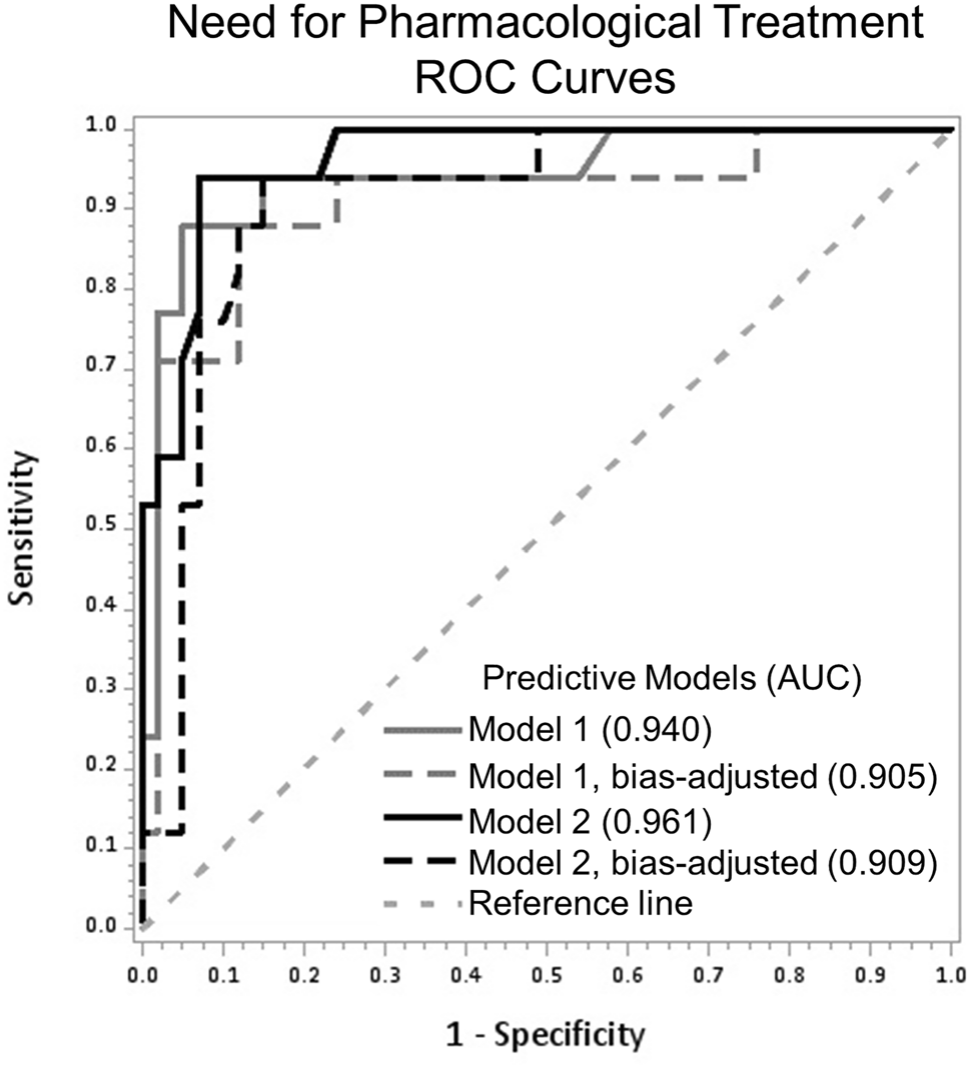
Receiver Operator Characteristic (ROC) Curves for Five miRNAs Differentiating *Pharmacologically*-*Treated* and *Not-Pharmacologically-Treated* Infants. ROC curves for performance of Model 1, containing miR-let-7d-5p, miR-128-3p, miR-30c-5p, miR-421, and miR-584-5p and Model 2, containing the same miRNAs and gestational age. *AUC* area under the ROC curve.

Predictive validity of each individual model miRNA is shown in Supplemental Table S4. The ROC curves for the signature miRNAs (Model 1) and miRNA with gestational age (Model 2), including bias-adjusted models, are shown in Fig. 2. The AUC were 0.94 and 0.96 for Models 1 and 2, respectively. Using a cutoff for predicted probability of ≥ 0.46, the overall accuracy of the miRNA signature was 93.1%, sensitivity was 88.2% (95% confidence interval [CI] 63.6; 98.5) and PPV was 88.2% (95% CI 63.6; 98.5) in unadjusted analyses (Table 5). After adjustment for gestational age, the overall accuracy remained at 93.1%, sensitivity was 94.1% (95% CI 71.3; 99.9) and PPV was 84.2% (95% CI 60.4; 96.6). Additional adjustment for the type of maternal MOUD (Model 3) resulted in similar predictions. As expected, AUC values and other validity indices for the bias-adjusted models were lower. Of the five miRNAs predictive of the need for pharmacological treatment, miR-128-3p, miR-421 and miR-584-5p were over-expressed and let-7d-5p and miR-30c-5p were under-expressed in *Pharmacologically-Treated* neonates compared to *Not-Pharmacologically-Treated* neonates.

**Table 5:** Predictive validity of the miRNA signature^a^ for identification of infants with NOWS requiring pharmacologic treatment or prolonged hospitalization.

Indices of validity^2^	Model 1			Model 2			Model 3		
	Firth Model^1^		Bias-adjusted	Firth Model^1^		Bias-adjusted	Firth Model^1^		Bias-adjusted
	Estimate	95% CI	Estimate	Estimate	95% CI	Estimate	Estimate	95% CI	Estimate
**Need for pharmacologic treatment**									
Accuracy, % correct of total	93.1		82.8	93.1		86.2	93.1		84.5
Sensitivity, %	88.2	(63.6; 98.5)	70.6	94.1	(71.3; 99.9)	82.4	88.2	(63.6; 98.5)	70.6
Specificity, %	95.1	(83.5; 99.4)	87.8	92.7	(80.1; 98.5)	87.8	95.1	(83.5; 99.4)	90.2
PPV, %	88.2	(63.6; 98.5)	70.6	84.2	(60.4; 96.6)	73.7	88.2	(63.6; 98.5)	75.0
NPV, %	95.1	(83.5; 99.4)	87.8	97.4	(86.5; 99.9)	92.3	95.1	(83.5; 99.4)	88.1
**Prolonged hospitalization**									
Accuracy, % correct of total	96.6		86.2	98.3		94.8	96.6		91.4
Sensitivity, %	93.3	(68.1; 99.8)	73.3	100.0	(78.2; 100.0)	93.3	100.0	(78.2; 100.0)	86.7
Specificity, %	97.7	(87.7; 99.9)	90.7	97.7	(87.7; 99.9)	95.3	95.3	(84.2; 99.4)	93.0
PPV, %	93.3	(68.1; 99.8)	73.3	93.8	(69.8; 99.8)	87.5	88.2	(63.6; 98.5)	81.3
NPV, %	97.7	(87.7,99.9)	90.7	100.0	(91.6; 100.0)	97.6	100.0	(91.3; 100.0)	95.2

Model 1 includes signature miRNAs.

Model 2 includes signature miRNAs and gestational age.

Model 3 includes signature miRNAs, gestational age, and the type maternal MOUD.

*CI* confidence interval, *MOUD* medication for opioid use disorder, *NPV* negative predictive value, *PPV* positive predictive value.

^a^Includes let-7d-5p, miR-128-3p, miR-30c-5p, miR-421, miR-584-5p for need for pharmacologic treatment and let-7b-5p, miR-10b-5p, miR-128-3p, miR-30c-5p, and miR-421 for prolonged hospitalization. For prolonged hospitalization, miR-10b-5p was dropped from Model 2 and miR-10b-5p, let-7b-5p, and miR-128-3p were dropped from Model 3 due to lack of convergence.

^1^Maximum likelihood estimates adjusted for small sample size using Firth's penalized likelihood approach.

^2^Based on using prediction probability point specified for each model (pharmacological treatment: Models 1 and 2: ≥ 0.46, Model 3: ≥ 0.60; prolonged hospitalization: Model 1: ≥ 0.55, Model 2: ≥ 0.45, Model 3: ≥ 0.40).

### Predictive validity of the miRNA signature for identification of infants requiring prolonged hospitalization

**Figure 3:**
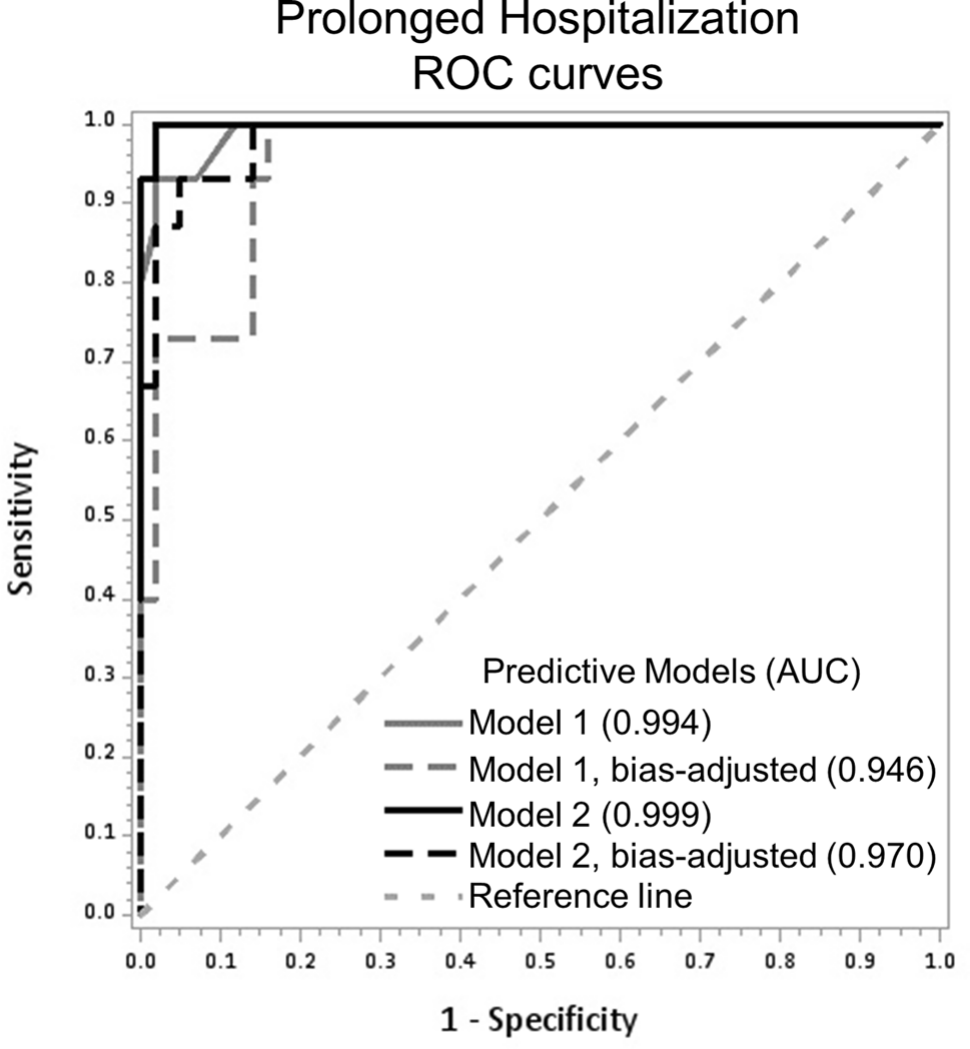
Receiver Operator Characteristic (ROC) Curves for Five miRNAs Differentiating Infants with Prolonged Hospitalization (≥ 14 Days) from Those Hospitalized for < 14 Days. ROC curves for performance of Model 1, containing let-7b-5p, miR-10b-5p, miR-128-3p, miR-30c-5p, and miR-421, and Model 2, containing the same miRNAs and gestational age. For Model 2, miR-10b-5p was dropped from the model due to lack of convergence. *AUC* area under the ROC curve.

While neonates in both groups experienced an extended hospital length of stay, a majority, i.e. 76.5%, of *Pharmacologically-Treated* neonates had a LOS ≥ 14 days whereas only 4.9% of neonates *Not-Pharmacologically-Treated* had a LOS ≥ 14 days (Table 3). The ROC curves for Models 1 and 2, including bias-adjusted models, are shown in Fig. 3. The AUC were 0.99 and 1.00 for Model 1 and Model 2, respectively. Using a cutoff for predicted probability of ≥ 0.55, the overall accuracy of the miRNA signature was 96.6%, sensitivity was 93.3% (95% CI 68.1; 99.8), and PPV was 93.3% (95% CI 68.1; 99.8) in unadjusted analyses (Model 1, Table 5). After inclusion of gestational age (Model 2) there was a lack of convergence (complete separation of the algorithm), and miR-10b-5p was dropped. For Model 2, overall accuracy was 98.3%, sensitivity was 100.0% (95% CI 78.2; 100.0) and PPV was 93.8% (95% CI 69.8; 99.8). Further inclusion of type of maternal MOUD exposure (Model 3) also resulted in non-convergence and complete algorithm separation and two additional miRNAs were dropped (let-7b-5p and miR-128-3p). The AUC was slightly lower in Model 3 (0.967), but overall accuracy was 96.6%. As expected, AUC values for the bias-adjusted models were lower, and other model measures were also lower when using the same cutoff for predicted probability (≥ 0.55). Of the five miRNAs predictive of prolonged hospitalization, miR-128-3p and miR-421 were over-expressed and let-7b-5p, miR-10b-5p, and miR-30c-5p were under-expressed in *LOS* ≥ *14 days* neonates compared to *LOS* < *14 days* neonates.

### Pathway enrichment analysis of miRNAs that predicted infant outcomes

Pathway enrichment analysis (for miRNAs_[PT,LOS]_, the three core miRNAs that predict both outcomes; miRNAs_[PT]_, the five miRNAs that predict need for pharmacological treatment; and miRNAs_[LOS]_, the five miRNAs that predict prolonged hospitalization) identified 11 biological pathways, based on Kyoto Encyclopedia of Genes and Genomes (KEGG) classification, that were significantly targeted by miRNAs from all three miRNA prediction groups, 9 pathways were significantly targeted by miRNAs from two of the three miRNA prediction groups, and 7 pathways that were significantly targeted by miRNAs in only one of the miRNA prediction groups (Supplemental Fig. S5). Of the 11 KEGG pathways significantly targeted by all three miRNA prediction groups, 3 were classified as addiction pathways, including morphine addiction.

## Discussion

The objective of this study was to identify circulating miRNAs obtained at birth from a readily available resource, infant cord blood, which were capable of *risk stratification of opioid-exposed infants* with respect to development of NOWS symptoms before those symptoms manifest. This study identified a signature of neonatal circulating miRNAs, detectable in umbilical cord blood plasma collected at birth, which predict severity of NOWS with more than 90% accuracy. Specifically, a panel of five miRNAs identified infants with severe NOWS requiring pharmacologic treatment with 88.2% sensitivity and 95.1% specificity. Similarly, five miRNAs (three overlapping with the predictive model for the need of pharmacologic treatment) accurately identified infants with prolonged hospitalization (93.3% sensitivity and 97.7% specificity). Moreover, even bias-adjusted models, used to reduce the influence of any one observation on model performance, resulted in overall accuracy of over 82%. Inclusion of gestational age at delivery and the type of maternal MOUD provided minimal improvement in the overall predictive performance of the miRNA panel. To our knowledge this is the first study reporting predictive utility of miRNA epigenetic markers for the risk stratification of infants born to women with OUD.

The predictive models identified in our cohort consist of multiple miRNAs, consistent with prior reports which indicate that circulating miRNAs have predictive value, and possibly serve biological functions, as a group rather than individually. For instance, we previously reported that miRNAs are altered in infants prenatally exposed to alcohol^38^, and that groups of miRNAs partially mediated effects of prenatal alcohol on infant growth and neurodevelopmental deficits. In another study, we also identified a group of miRNAs elevated at mid-pregnancy in pregnant women with heavy alcohol exposure, whose infants were affected by that exposure, which collectively explained between 24 and 31% of the variation in infant growth parameters at birth^35^. Moreover, these elevated maternal miRNAs collectively impaired fetal and placental growth in pregnant mice, and collectively, but not individually, inhibited growth of human placental cells in vitro, suggesting molecular synergy^37^. We have also found that plasma miRNAs collected from pregnant women were differentially expressed among women with OUD, women with alcohol exposure during pregnancy, and unexposed controls^34^. These data collectively support the hypothesis that plasma miRNAs are affected by prenatal drug exposure and mediate effects of prenatal drug exposure on infant outcomes. Yet, care should be taken against the causal inference between prenatal opioid exposure and changes in miRNA expression, but instead the profile of miRNAs that are predictive of NOWS likely reflect changes across biological systems that contribute to both risk and resilience to the development of NOWS.

Interestingly, while the three miRNAs in the final signature were predictive of both NOWS outcomes, need for pharmacologic treatment and length of stay, two additional miRNAs were identified that were specific for each outcome, suggesting that biological mediators of each outcome are likely to be similar, though not completely overlapping. When miRNA models were restricted to the three miRNAs predictive of both NOWS outcomes, the accuracy, AUC, and PPV were good, but all values improved with the addition of the two outcome-specific miRNAs and sensitivity was greatly improved. In this context, within the larger set of 51 miRNAs which met initial eligibility criteria, the majority (n = 26) were predictive of both outcomes, though outcome-specific miRNAs were also identified. It should be noted that among infants with prolonged hospitalization (n = 15) the majority required treatment (86.7%) and among the 26 miRNAs predictive of both outcomes, effect sizes were larger for prolonged hospitalization. Both the need for pharmacologic treatment and prolonged hospitalizations are the commonly used measures of NOWS severity^39,40^; however, there is currently, no ‘gold standard’ definition of NOWS, and substantial variability in clinical scoring tools and treatment protocols^41,42^. NOWS symptoms of maladaptation are highly variable and typically manifest as physiologic and/or behavioral dysregulation in four key domains—state control/attention, motor and tone control, sensory processing, and autonomic control^27^. While we hypothesize that miRNAs predictive of both outcomes might constitute the most robust signature, variability in miRNA expression may be influenced by the dominance of specific NOWS phenotypes, a possibility that requires further investigation.

Previous studies primarily focused on the role of maternal MOUD, polysubstance use, infant sex, gestational age at birth, and use of psychotropic medication on NOWS severity^29,43–45^. A more limited number of studies have evaluated the role of epigenetic factors, such as DNA methylation and miRNA expression. Epigenetic analysis indicates that CpG-rich regions in the promotor for the µ opioid receptor (*OPRM1*) are hypermethylated following acute exposure to prescription opioids^46^, in individuals with OUD^47,48^, and following methadone-maintenance therapy^49,50^. Likewise, neonates prenatally exposed to maternal methadone have higher methylation across assessed promotor and exon-1 regions of the *OPRM1 gene* than non-opioid exposed neonates^51^ and increased *OPRM1* promoter methylation has been associated with the need for two or more medications for pharmacological intervention for NOWS^52^. miRNAs can also impact *OPRM1* expression and, reciprocally, exhibit altered expression due to opioid exposure. Of the candidate miRNAs identified in our study, 13 (25.5%) have been previously implicated as targeting *OPRM1*^53,54^ and 22 (43.1%) have been previously shown to have altered expression following opioid exposure^33,53–57^. Interestingly, both groups in our study were opioid-exposed, and therefore, any differences in miRNA expression between neonates pharmacologically treated for NOWS and non-treated infants cannot be attributable solely to the activation of opioid signaling pathways by MOUD. Yet, the candidate miRNAs included three members of the let-7 miRNA family, which target *OPRM1*^53,54,58^ and increase morphine tolerance through reduced MOR protein expression^58^. Therefore, these altered miRNAs may alter biological responses to MOUD exposure, including tolerance, thereby exacerbating or prolonging maladaptive responses to MOUD exposure.

A recent study examined changes to placental DNA methylation that occur in response to maternal OUD and may be predictive of NOWS development^59^. None of the miRNAs identified in our study (Table 4) had differential DNA methylation at their genomic loci when comparing placenta from POE neonates who would develop NOWS to neonates who did not develop NOWS. One CpG site (cg19782652) in promoter/enhancer regions upstream of let-7e and miR-125b was hypomethylated in POE neonates who develop NOWS when compared to POE-naïve neonates. Interestingly, both let-7e and miR-125b met criteria for inclusion in the model to predict prolonged hospitalization, and both also exhibited decreased expression in neonates with LOS ≥ 14 days. These data indicate that the placenta may play a role in modulating the expression of candidate plasma miRNAs present in umbilical cord blood.

The miRNAs in circulation we identified as predictive of both need for pharmacologic treatment and prolonged hospitalization, miR-421, miR-128-3p, and miR-30c-5p have been previously implicated in modulating inflammatory tone. For instance, miR-421 was increased in human leukocytes following environmental particulate-exposure and its elevation correlated with altered expression inflammatory signaling pathway genes^60^. In human bone mesenchymal cells, miR-128-3p overexpression exacerbated tumor necrosis factor-α-induced pro-inflammatory cytokine release^61^, and overexpression of miR-30c-5p in a human macrophage cell line was associated with decreased release of pro-inflammatory cytokine, IL-1β^62^. Moreover, in our previous work examining infant circulating miRNA expression following prenatal alcohol exposure, miR-30c-5p clustered with a group of miRNAs that targeted the immune system and inflammation and, as a group, partially mediated the effects of prenatal alcohol exposure on infant length and neurodevelopment^38^. POE itself is documented to increase inflammatory cytokines and potentiate response to immune challenges in a rat model^7^. Here, both miR-421 and miR-128-3p were significantly increased, while miR-30c-5p was significantly decreased in cord blood plasma of neonates with worse outcomes which is consistent with a hypothesis that these miRNAs are associated with a pro-inflammatory state that may predict the onset of NOWS.

While miRNAs in the plasma can come from multiple tissues, thereby reflecting the biological state of many different cells, we have previously found that miRNAs within the plasma do act in concert to control biological outcomes^37,38^. Using pathway enrichment analysis, we found that miRNAs in all three prediction groups, miRNA_[PT,LOS]_, miRNAs_[PT]_, and miRNAs_[LOS],_ target gene transcripts associated with generalized addiction. Additionally, miRNAs_[LOS]_ target transcripts associated with intestinal barrier integrity and function (e.g. mucin type o-glycan synthesis^63^, extracellular matrix-receptor signaling^64^, amoebiasis^65^, focal adhesion and PI3K-Akt signaling^66^), and miRNAs_[PT]_ target transcripts associated with immunomodulation (e.g. TGF-β signaling^67^, *N*-glycan biosynthesis^68^, retrograde endocannabinoid signaling^69^). These associations require further testing, particularly the pathways targeted could be organ/tissue dependent or may not be the primary site of action for the altered miRNAs. Furthermore, we do not as yet have enough information to understand the biology of these miRNAs. However, previous studies found that some miRNAs that are elevated in disease pathologies are protective while others are maladaptive^37^ and we expect the same outcome is likely to be true with NOWS-predictive miRNAs.

From a clinical perspective, risk stratification in infants with prenatal opioid exposure before manifestation of NOWS symptoms is of great importance. Per national guidelines, infants born to women who used opioids during pregnancy are monitored for at least 96 h in the hospital for signs of NOWS^15^. Current clinical tools guiding treatment decisions rely on the presence of withdrawal signs, which typically manifest by day 4 and are common on days 1 and 2 after birth, and are thus reactive in nature. Proactive identification of low-risk infants with high degree of accuracy will allow clinicians to shorten observation periods. Shorter hospitalization will not only result in healthcare cost savings but will improve maternal-infant bonding during the first days after birth—a crucial protective factor for long-term neuro-behavioral outcome in substance-exposed infants^70,71^. Identification of newborns at lower risk for needing pharmacological treatment might allow rural and community hospital to keep more of these infants in their postpartum units avoiding costly and socially disruptive transfer of these infants to tertiary care hospitals. A 2017 national survey demonstrated that while observation for NOWS is common in Level 1 nurseries, 87% of the Level 1 nurseries transferred to NICUs when pharmacological treatment was indicated^72^. On the other hand, identification of high-risk infants by the rapid laboratory assessment tool within the first hours of life will inform decisions about most appropriate clinical monitoring and access to care (e.g., timely transfer of high-risk infants from rural/underserved areas to tertiary care facilities) as well as supporting proactive intervention approaches. Increased attention to non-pharmacological interventions for high-risk infants including additional breastfeeding support when appropriate, and consideration of a lower threshold for pharmacological treatment are potential responses for infants identified as high risk if the miRNA results may be obtained within the first few days of life.

Several strengths of the study also should be mentioned. First, a prospective cohort design allowed for collection of detailed information on substance use (MOUD, illicit drugs, alcohol, tobacco) by repeated prospective interviews and urine drug panels. Secondly, miRNAs were assayed non-invasively and using tissues that would otherwise be waste tissues. Thirdly, miRNA assessment by qPCR is a transferrable technology that is already employed in clinical settings. Moreover, this screen identified a number of abundant plasma miRNAs that can be further investigated for their role as mediators of adaptive biology, as we have reported for other adverse pregnancy outcomes^37^. Fourth, we examined the effect of the strongest confounders—maternal type of MOUD and gestational age at delivery, as well as applied a bias reduction approach in statistical modelling when estimating validity indices of signature miRNAs. Fifth, our cohort included a high percentage of Latinx population, reflecting the general population of New Mexico, addressing an unmet need to better understand the impact of prenatal MOUD exposure and NOWS in the Latinx community which has historically been underrepresented in studies of this nature.

Our findings should be weighed within the context of several study limitations. First, the sample size is relatively modest, and study findings will require validation in other population. Study findings can only be generalized to infants prenatally exposed to MOUD (with or without other opioids), while prenatal exposure to other opioids without MOUD may result in a different circulating miRNA signature. Moreover, further validation studies are needed to characterize the impact of maternal MOUD type, i.e., buprenorphine or methadone, on the accuracy of miRNAs in NOWS risk stratification. Second, polysubstance use is a common phenomenon in women with MOUD, and while every effort has been made to carefully control for the effect of co-exposures (e.g., exclusion of stimulant co-exposures, careful quantification of other substances), residual confounders cannot be ruled out. It should be noted though, that the prevalence of co-exposures was similar among the study groups, though types of co-exposures and methods of opioid use may impact the miRNA profile and biomarker sensitivity and specificity. Future larger studies are warranted in populations with polysubstance use to assess the stability of the miRNAs identified here in those populations especially in the context of prevalent co-exposures, such as tobacco^73,74^, and the rising use of methamphetamines among pregnant women with opioid use disorder^75^. Third, similar to other studies in the field^76,77^, we have excluded infants born before 34 weeks of gestation, but included 6 preterm infants (two at 34 weeks, four at 36 weeks). The length of hospitalization is particularly sensitive to gestational age at delivery, but here we found that for these 6 infants, outcomes were still variable, as two did not receive pharmacological treatment and one had a length of stay < 14 days. Additionally, there is increasing recognition that gestational age, while being the most commonly adjusted for covariate in obstetrics and perinatal epidemiology, might be causally linked to outcomes^78–80^. Therefore, analyses were conducted before and after adjustment for gestational age. However, models with adjustment for gestational age, and additional adjustment for type of maternal MOUD exposure, performed only slightly better than models without. Accuracy of the prolonged hospitalization model using miRNA alone was 96.6% and inclusion of gestational age and MOUD resulted in a surplus of predictive information as indicated by the lack of model convergence. We also recognize that some signs of prematurity and NOWS (e.g. respiration difficulties, poor feeding) might be hard to distinguish clinically, and common epigenetic mechanisms might play in role in both conditions. Future studies should examine the predictive ability of miRNAs in larger samples of full-term, late preterm, and early preterm infants. Fourth, while miRNAs were assayed using clinically-relevant qPCR technology, there are advantages to performing genome-wide RNA sequencing, including the quantification of the entire miRNA transcriptome and the identification of splice variants. Therefore, additional work using sequencing to identify miRNA biomarkers could provide additional insight into miRNAs that are predictive of NOWS.

In summary, this is the first proof-of-principle report laying out the foundation for the utilization of miRNAs derived from umbilical cord at birth for risk stratification of opioid-exposed infants. While validity indices of the identified miRNA panel are very encouraging, results should be interpreted with caution before validation in different study populations. Potentially, miRNA panels might need to be developed for different patient subpopulations with scoring algorithms taking into considerations results of miRNA analysis and key clinical indicators (e.g., gestational age at delivery, maternal exposures, known comorbidities).The ability to identify infants with MOUD exposure that are at lower and high risk for prolonged LOS and pharmacological treatment can result in improved utilization of health care resources and minimize maternal-infant separation that often accompanies transfer to tertiary care hospitals.

## Methods

### Study design and population

This analysis used a study population derived from the ‘*Ethanol, Neurodevelopment, Infant and Child Health*’ (ENRICH) prospective cohort study, with recruitment and sample collection from March 2015 to November 2019. The primary objective of the parent ENRICH study was to identify early indices of functional brain damage associated with prenatal alcohol exposures^5,6,81^. In addition to unexposed controls, patients receiving MOUD were recruited as another comparison group due to similarities in pre- and post-natal environment between substance-using populations. The cohort was formed by recruiting pregnant women during one of the first prenatal care visits. Children born to cohort participants were followed for up to 20 months of age. The ENRICH study included 4 prospective visits, and data obtained from Visit 1 (prenatal) and Visit 2 (delivery/birth) were used in this analysis. The general eligibility criteria for the ENRICH study were: (1) ≥ 18 years old; (2) singleton pregnancy confirmed by ultrasound; (3) no fetal diagnosis of a major structural anomaly; (4) no more than minimal use of cocaine, crack-cocaine, MDMA (ecstasy), or methamphetamine use (per repeated prospective self-reports and repeated urine drug screen (UDS) tests). Minimal use is defined as no more than 1 urine drug test or incidence of self-report during the first trimester and abstention from use of these substances during the second and third trimesters^5^. For this analysis, the following additional eligibility criteria were employed: (1) participants recruited into MOUD group; (2) no self-reported alcohol use in the third trimester and no more than one positive ethanol biomarker in a comprehensive panel (described below); (3) gestational age at delivery ≥ 34 weeks; (4) available umbilical cord blood sample.

Human subjects’ approval was obtained from the University of New Mexico Human Research Review Committee and Texas A&M University Institutional Review Board. All participants provided written informed consent. All procedures were followed according to the relevant guidelines.

### Characterization of maternal MOUD and other prenatal factors

The MOUD group was recruited from the University of New Mexico Milagro Clinic, an interdisciplinary clinic specializing in treating pregnant women with substance use disorders. The objective was to identify miRNAs capable of risk stratification of opioid-exposed infants with respect to development of NOWS symptoms rather than to identify miRNAs associated with prenatal opioid exposure. Therefore, the current study did not include unexposed infants. The type of MOUD (methadone vs. buprenorphine) and dose before delivery were abstracted from electronic medical records. Co-exposure with illicit drugs was assessed throughout pregnancy (timing and frequency) by prospective repeated interviews, based on the 2011 National Survey on Drug use and Health^82^. Self-reported information was verified by study-specific Urine Testing Drug Panel-7 (UDS-7; US Drug Testing Lab, Des Plaines, IL) conducted at Visit 1 and Visit 2. The UDS-7 panel measured amphetamines, barbiturates, benzodiazepines, cocaine, opiates, PCP, and cannabinoids/THC metabolites. Alcohol use was captured by three timeline follow-back (TLFB) interviews which captured use a month around conception, 30 days before enrollment, and 30 days before delivery. Quantity and frequency of reported alcohol at each day were converted into the ounces of absolute alcohol per day (AA/day^83^) and averaged across TLFB calendars. In addition, a panel of ethanol biomarkers (gamma-glutamyl transpeptidase [GGT, > 40U/L], carbohydrate deficient transferrin [%dCDT, > 2.0%], phosphatidylethanol [PEth, > 8 ng/mL], urine ethyl glucuronide [uEtG, > 38 ng/mL] and ethyl sulfate [uEtS, > 25 ng/mL]) was evaluated in maternal samples at baseline and delivery visits as well as PEth [> 24.9 ng/mL] measured in dry blood spots of a newborn^84–86^.

### NOWS severity measures, infant and postnatal environment measures

The primary outcomes of interest were to identify miRNAs that predict: (1) which opioid-exposed neonates will be *Pharmacologically-Treated,* compared to neonates *Not-Pharmacologically-Treated,* and (2) which opioid-exposed neonates will have an extended length of hospital stay, defined as a *LOS* ≥ *14 days* compared to neonates with *LOS* < *14 days*. These measures are widely used in clinical studies for assessment of NOWS severity^42,87,88^. According to the University of New Mexico Hospital (UNMH) guidelines, pharmacological treatment of NOWS was initiated if an infant scored ≥ 8 on the Finnegan Neonatal Abstinence Scoring Tool^89^ on three consecutive assessments, if the mean of three scores is ≥ 8, or if two consecutive scores are ≥ 12^90^, or was initiated based on the Eat, Sleep, and Console evaluation model. Newborns are scored at admission (~ 2 h of age). All infants with confirmed or suspected prenatal opioid exposure remain inpatient at UNMH for a minimum of 96 h.

### Circulating miRNA analyses

#### Umbilical cord blood collection and RNA isolation

Immediately following delivery, blood samples were collected from umbilical cord in EDTA-coated tubes. Plasma was separated by centrifugation at 2000*g* for 10 min, collected in 200 µL aliquots, placed at − 80 °C, shipped to Texas A&M University on dry ice, and stored at − 80 °C until processing. Total RNA was isolated from 125 to 200 µL of plasma using the miRNeasy mini kit (Qiagen Sciences, Germantown, MD) with the addition of 1.2 µg carrier MS2 phage RNA (Roche Diagnostics, Mannheim, Germany), according to manufacturer’s protocols. RNA was eluted using 20 µL nuclease-free water and stored at − 80 °C until cDNA synthesis.

### cDNA synthesis and qPCR

cDNA was synthesized from 6.25 ng RNA, diluted to 5 ng/µL using nuclease free water, in a 10 µL cDNA reaction mixture using the miRCURY LNA RT Kit (Qiagen Sciences, Germantown, MD). At the time of cDNA synthesis, spike-in miRNAs cel-miR-39-3p and UniSp6 (RNA Spike-In Kit, for RT, Qiagen Sciences, Germantown, MD) were added to the reaction mixture per the manufacturer’s directions to allow for the detection of potential RT and PCR inhibitors. cDNA was diluted 110× and combined with an equal amount of 2× miRCURY SYBR Green Master Mix (Qiagen Sciences, Germantown, MD). Two independent samples were loaded onto the 384-well miRCURY LNA miRNA Human Serum/Plasma Focus miRNA PCR Panels (YAHS-106Y, Qiagen Sciences, Germantown, MD) and qPCR was performed on the 7900HT Fast Real-Time PCR System (Applied Biosystems, Foster City, CA). Two additional proof-of-principle samples had been previously assessed using miRCURY LNA Human miRNome panels v3. miRNA expression values were extracted (1) for primers which were unchanged across panel versions and (2) primers that were altered across versions but had comparable amplification to the Focus panel Supplementary information. Cycle threshold (CT) and target amplification were assessed using SDS 2.4 software (Applied Biosystems/Thermo Fisher Scientific Waltham, MA). We used qPCR to assay plasma miRNA expression as it has high specificity and sensitivity compared to other methods of miRNA analysis and is considered the ‘gold standard’ technique to validate sequencing and microarray studies^91–93^. We have previously found excellent correlation of expression of miRNAs with assay replication in the same biological samples, indicating the reliability of this qPCR protocol^94^.

### Assessment of erythrocyte contamination of plasma RNA

Plasma samples were assayed to assess potential erythrocyte contamination as previously described^35,36,38^, since lysed erythrocytes are a contaminating source of miRNAs. Spectrophotometric analysis of plasma was used to detect the presence of free hemoglobin, with absorbance at 414 nm > 0.3 as an indicator of possible hemolysis. Following RNA isolation, expression of erythrocyte-enriched transporter SLC4A1/BAND3 was assessed using the qScript cDNA Synthesis Kit (Quantabio, Beverly, MA) and PerfeCTa SYBR Green FastMix (Quantabio, Beverly, MA), and enrichment of erythrocyte miRNA was determined by comparing the expression of erythrocyte-enriched miR-451 to the expression of miR-23a which is stably-expressed in plasma (ΔCT_hemolysis_ = CT_miR-23a_ − CT_miR-451_)^95^. A ΔCT_hemolysis_ of > 7 has been found to be a positive indicator of hemolysis^95^. In samples where SLC4A1/BAND3 was below the limit of detection for qPCR, we used absorbance at 414 nm and ΔCT_hemolysis_ in tandem as an additional indicator of hemolysis due to previous findings that in infants absorbance alone is not a reliable hemolysis marker^38^, likely due to the conversion of fetal to adult hemoglobin.

### Data analysis

#### Exclusion of cord blood samples

66 umbilical cord samples were collected from infants with prenatal maternal MOUD exposure. One sample was excluded due to insufficient volume available for analysis. Six were excluded due to potential hemolysis, with three samples positive for the erythrocyte transporter SLC4A1/BAND3 and three samples that were SLC4A1/BAND3 negative but positive for both the additional hemolysis markers absorbance at 414 nm (> 0.3) and a ΔCT_hemolysis_ (> 7; see Supplementary Fig. S2). miRNA expression in the two proof-of-principle samples was compared miRNA expression for all Focus panel samples. One proof-of-principle sample was determined to be an outlier based on principal component analysis of miRNA expression and was excluded. Resultant sample size of 58 subjects was used in the analysis.

#### Data processing and modifications

CTs were assessed for each miRNA. CT values were normalized to the sample global mean of expression for all detected miRNAs (ΔCT) for each sample. We further examined miRNAs which were expressed in > 80% of samples within a group. miRNAs with undetected CTs were considered expressed below the detectable limit and, therefore, were assigned at CT value 1 unit above the highest CT for that miRNA, as assigning these values has been shown to reduce bias^96^. Comparisons of normalized miRNA expression (ΔCT) between outcome groups is denoted as ΔΔCT and 1 ΔΔCT approximates a two-fold change in expression between groups. Out of 179 assessed miRNAs, 171 miRNAs were expressed in > 80% of the samples, had consistent amplification across panels, and were further analyzed.

#### Pathway enrichment analysis

We performed pathway enrichment analysis to determine possible biological pathways that are targeted by three clusters of miRNAs: miRNAs_[PT,LOS]_, the three core miRNAs that predict both outcomes; miRNAs_[PT],_the five miRNAs that predict need for pharmacological treatment; and miRNAs_[LOS]_, the five miRNAs that predict prolonged hospitalization. Using DIANA-mirPath v3.0^97^, mRNA targets of the selected groups of miRNAs were determined using the in silico target prediction tool DIANA-microT-CDS v5.0 and pathways were identified that had significant overrepresentation for targeted mRNA transcripts. We used the KEGG analysis pipeline with the following parameters: microT threshold of 0.8, p-value > 0.01; and FDR correction. Briefly, this tool uses Fisher’s exact test and unbiased empirical distributions to determine if a miRNA has enriched pathway targeting compared to other annotated miRNAs and, subsequently, uses meta-analysis statistics to determine the pathways targeted by a set of miRNAs^97^.

### Statistical approaches

Univariate comparisons were conducted using, as appropriate, t-test or Wilcoxon rank-sum test for continuous variables and Chi-square test or Fisher’s exact test for categorical variables. Comparisons between infants *Pharmacologically-Treated* and infants *Not-Pharmacologically-Treated* were conducted for maternal and infant sociodemographic and clinical factors and maternal substance use. Unadjusted comparisons (Student’s t-test) of miRNA expression were conducted for the outcomes *Pharmacologically-Treated* vs *Not-Pharmacologically-Treated* and *LOS* ≥ *14 days* vs *LOS* < *14 days*. Effect sizes (Cohen’s *d*^98^) were calculated to compare miRNA expression between outcome groups overall and stratified by type of MOUD. A moderate effect size of *d* > 0.40 is indicative of clinically relevant alterations in expression^99,100^.

Predictive models for each of the outcomes were constructed by including miRNAs meeting the following criteria: (1) *d* > 0.40 for mean difference in miRNA expression in unadjusted analyses; (2) p < 0.10 for the differences in miRNA expression. The final ‘miRNA signature’ parsimonious models were developed for each outcome using step-wise logistic regression (Model 1)^101^. Firth’s penalized likelihood approach was used to determine model predicted probabilities as it controls for potential regression model lack of convergence, or separation, related to the small sample size^102,103^. In the event that complete separation of the model still occurred (indicating an overspecified model or not enough data given the predictors), the least significant miRNA was dropped from the model^104^.

Since the sample size precluded having separate algorithm development and validation models, a second model applied a bias reduction method that estimated the predicted probability for a given observation by approximating the exclusion of that given observation^105^. This adjustment to model fit is provided as ‘bias-adjusted’ estimates. ROC curves were constructed and AUC was estimated. Final predictive models were adjusted for (1) gestational age (Model 2) and (2) gestational age and type of maternal MOUD (Model 3). A predicted probability cut-off point was selected optimizing sensitivity and specificity^106^. Sensitivity, specificity, PPV, and NPV and associated 95% confidence intervals (95% CI) are reported. All statistical analyses were conducted using R^107^ and SAS 9.4 (Cary, NC).

## Supplementary Information

Supplementary Information.

Supplementary Table S3.

## Data Availability

Mean expression difference, effect size, and significance for all miRNAs analyzed for this study are included in the supplementary information files (see Supplementary Table S3). Raw miRNA qPCR amplification data generated and/or analyzed during the current study are not publicly available due the lack of data sharing acknowledgement of de-identified data in the consent and IRB protocol. The request for data sharing can be considered on the case-by-case basis with a formal data sharing agreement between institutions.

## Abbreviations

AA: Absolute ounces of alcohol
AUC: Area under the curve
CT: Cycle threshold
LOS: Length of stay
MIMAT: MiRbase accession number for mature miRNA
miRNA: MicroRNA
miRNA_[LOS]_: MiRNAs predictive of prolonged length of hospital stay
miRNA_[PT]_: MiRNAs predictive of need for pharmacological treatment for NOWS
miRNA_[PT,LOS]_: MiRNAs predictive of both prolonged length of hospital stay and need for pharmacological treatment
MOUD: Medication for opioid use disorder
NOWS: Neonatal opioid withdrawal syndrome
NPV: Negative predictive value
OUD: Opioid use disorder
PPV: Positive predictive value
ROC: Receiver operator characteristic
TLFB: Timeline follow-back
UDS: Urine drug screen
