# Supplementary material for: Prenatal opioid-exposed infant extracellular miRNA signature obtained at birth predicts severity of neonatal opioid withdrawal syndrome
Source: Sci Rep. 2022 Apr 8;12:5941. doi: 10.1038/s41598-022-09793-7 (PMC8993911; doi:10.1038/s41598-022-09793-7)
Supplement: Supplementary file 1 — Supplementary Information. [file 41598_2022_9793_MOESM1_ESM.pdf]

Prenatal Opioid-Exposed Infant Extracellular miRNA Signature Obtained at Birth Predicts Severity of Neonatal Opioid Withdrawal Syndrome

Amanda H. Mahnke[1*], Melissa H. Roberts[2], Lawrence Leeman[3,4], Xingya Ma[2], Ludmila N Bakhireva[2,3,5#], Rajesh C. Miranda[1#]

[*]Corresponding author

[#]Equally contributing senior authors

[1]Texas A&M University Health Science Center, Department of Neuroscience and Experimental Therapeutics, Bryan, TX, 77807 USA

[2]University of New Mexico College of Pharmacy, Department of Pharmacy Practice and Administrative Sciences, Substance Use Research and Education (SURE) Center, Albuquerque, NM, 87131 USA

[3]University of New Mexico School of Medicine, Department of Family and Community Medicine, Albuquerque, NM, 87106 USA

[4]University of New Mexico School of Medicine, Department of Obstetrics and Gynecology, Albuquerque, NM, 87106 USA

[5]University of New Mexico School of Medicine, Department of Internal Medicine, Division of Epidemiology, Biostatistics and Preventive Medicine, Albuquerque, NM, 87106 USA

Supplementary Table S1: **miRNA Primers Altered between Panel Versions for Proof-of-Concept Samples**

miRNA	MIMAT	Analysis Status[1]
hsa-miR-17-5p	MIMAT0000070	Exclude
hsa-miR-26a-5p	MIMAT0000082	Include
hsa-miR-27a-3p	MIMAT0000084	Include
hsa-miR-30d-5p	MIMAT0000245	Exclude
hsa-miR-181a-5p	MIMAT0000256	Include
hsa-miR-223-3p	MIMAT0000280	Include
hsa-miR-128-3p	MIMAT0000424	Include
hsa-miR-132-3p	MIMAT0000426	Include
hsa-miR-143-3p	MIMAT0000435	Include
hsa-miR-126-5p	MIMAT0000444	Include
hsa-miR-185-5p	MIMAT0000455	Include
hsa-miR-186-5p	MIMAT0000456	Include
hsa-miR-320a	MIMAT0000510	Include
hsa-miR-99b-5p	MIMAT0000689	Include
hsa-miR-361-5p	MIMAT0000703	Include
hsa-miR-331-3p	MIMAT0000760	Exclude
hsa-miR-339-5p	MIMAT0000764	Include
hsa-miR-335-5p	MIMAT0000765	Include
hsa-miR-133b	MIMAT0000770	Include
hsa-miR-451a	MIMAT0001631	Include
hsa-miR-485-3p	MIMAT0002176	Include
hsa-miR-495-3p	MIMAT0002817	Include
hsa-miR-574-3p	MIMAT0003239	Exclude
hsa-miR-320b	MIMAT0005792	Include

[1] miRNA primers which did not have similar performance between panel versions were excluded from subsequent analyses

MIMAT, miRBase accession number for mature miRNA

Supplementary Figure S2: Hemolysis, RNA Isolation, and Panel Performance Metrics

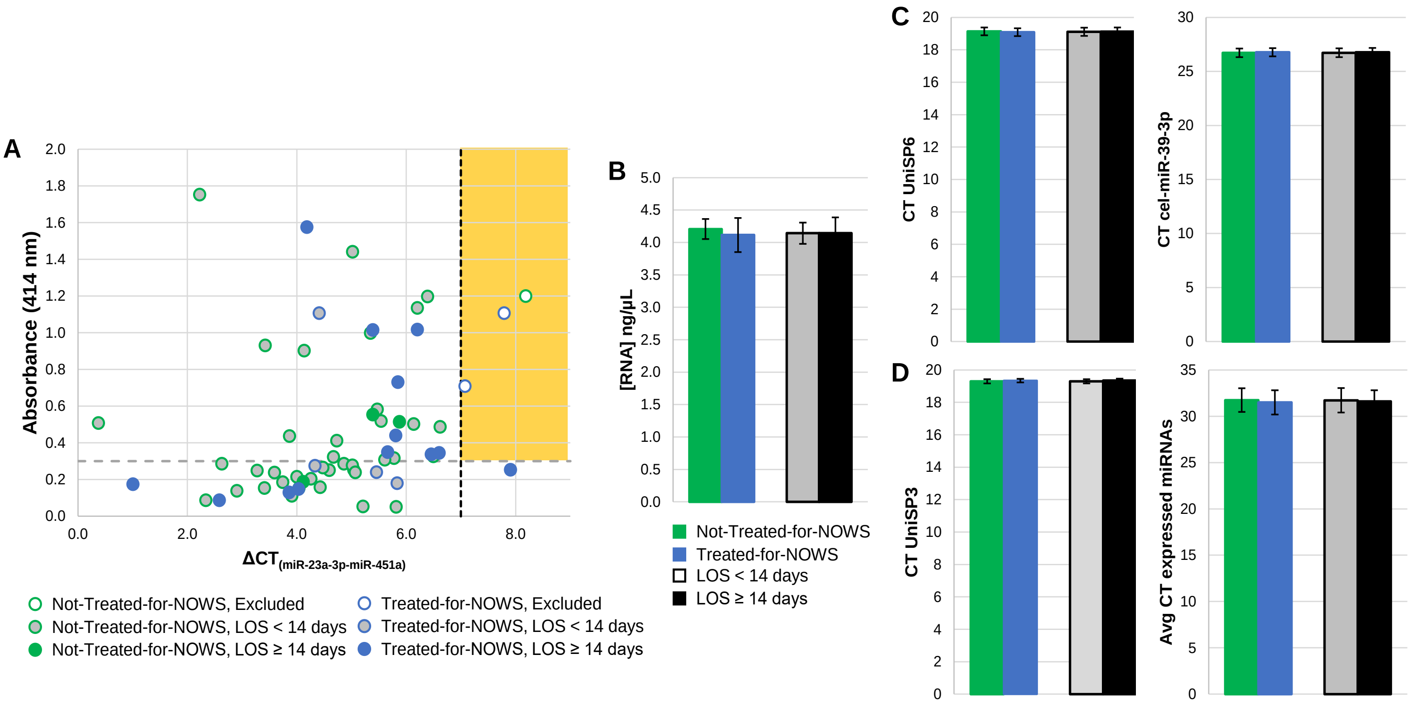

Supplementary Figure S2: (A) Comparison of hemolysis detection metrics of absorbance at 414 nm and expression of erythrocyte-enriched miRNA ($\Delta CT_{(miR\text{-}23a\text{-}3p\ –\ miR\text{-}451a)}$). Yellow region indicates samples which were positive for both hemolysis measures and excluded from further analysis. (B) Total RNA concentration in plasma samples including the addition of carrier MS2 phage RNA. (C) Expression of spike-in miRNAs to detect reverse transcriptase and polymerase inhibition. (D) Panel performance metrics including expression of interpolate control (UniSP3) and average CT of expression for miRNAs.

Supplemental Table S4: Predictive Model Construction and Performance

Need for pharmacological treatment models

	Model 1			Model2			Model 3		
	Firth Model[1]		Bias-adjusted	Firth Model[1]		Bias-adjusted	Firth Model[1]		Bias-adjusted
Logistic Regression Model	**Estimate**	**P-value**	**Estimate**	**Estimate**	**P-value**	**Estimate**	**Estimate**	**P-value**	**Estimate**
AUC, estimate	0.940		0.905	0.961		0.909	0.971		0.904
Factors, parameter estimates (SE)									
hsa-let-7d-5p	-1.32 (0.59)	0.03		-1.24 (0.64)	0.05		-1.03 (0.62)	0.10	
hsa-miR-128-3p	2.19 (0.89)	0.01		1.24 (0.93)	0.19		0.92 (0.89)	0.30	
hsa-miR-30c-5p	-2.08 (1.12)	0.06		-2.33 (1.32)	0.08		-2.16 (1.32)	0.10	
hsa-miR-421	0.83 (0.58)	0.15		0.79 (0.58)	0.17		0.87 (0.58)	0.13	
hsa-miR-584-5p	1.83 (0.81)	0.02		1.42 (0.91)	0.12		1.13 (0.85)	0.18	
gestational age (weeks)	----			-0.73 (0.41)	0.07		-0.66 (0.41)	0.10	
methadone vs buprenorphine	----			----			1.15 (0.88)	0.19	

	Firth Model[1]		Bias-adjusted	Firth Model[1]		Bias-adjusted	Firth Model[1]		Bias-adjusted
Predictions from Model[2]	**Estimate**	**95% CI**	**Estimate**	**Estimate**	**95% CI**	**Estimate**	**Estimate**	**95% CI**	**Estimate**
Prediction Probability Point	≥ 0.46			≥ 0.46			≥ 0.60		
Pharm treatment, correct	15		12	16		14	15		12
Pharm treatment, incorrect	2		5	3		5	2		4
No Pharm treatment, correct	39		36	38		36	39		37
No Pharm treatment, incorrect	2		5	1		3	2		5
Accuracy, % correct of total	93.1		82.8	93.1		86.2	93.1		84.5
Sensitivity, %	88.2	(63.6;98.5)	70.6	94.1	(71.3;99.9)	82.4	88.2	(63.6;98.5)	70.6
Specificity, %	95.1	(83.5;99.4)	87.8	92.7	(80.1;98.5)	87.8	95.1	(83.5;99.4)	90.2
Positive predictive value, %	88.2	(63.6;98.5)	70.6	84.2	(60.4;96.6)	73.7	88.2	(63.6;98.5)	75.0
Negative predictive value, %	95.1	(83.5;99.4)	87.8	97.4	(86.5;99.9)	92.3	95.1	(83.5;99.4)	88.1

Prolonged hospitalization predictive model

	Model 1			Model 2			Model 3		
	Firth Model[1]		Bias-adjusted	Firth Model[1]		Bias-adjusted	Firth Model[1]		Bias-adjusted
Logistic Regression Model	**Estimate**	**P-value**	**Estimate**	**Estimate**	**P-value**	**Estimate**	**Estimate**	**P-value**	**Estimate**
AUC, estimate	0.994		0.946	0.999		0.970	0.991		0.966
Factors, parameter estimates (SE)									
hsa-let-7b-5p	-3.46 (1.45)	0.02		-3.57 (1.56)	0.02		----		
hsa-miR-10b-5p	-1.29 (0.89)	0.15		----			----		
hsa-miR-128-3p	2.86 (1.48)	0.05		4.16 (2.33)	0.07		----		
hsa-miR-30c-5p	-8.06 (3.49)	0.02		-6.63 (3.30)	0.04		-3.71 (2.09)	0.08	
hsa-miR-421	3.25 (1.42)	0.02		1.95 (1.04)	0.06		3.35 (1.47)	0.02	
gestational age (weeks)	----			-1.36 (0.66)	0.04		-1.67 (0.58)	0.004	

methadone vs buprenorphine	----			----			3.82 (1.71)	0.03	
	**Firth Model[1]**		**Bias-adjusted**	**Firth Model[1]**		**Bias-adjusted**	**Firth Model[1]**		**Bias-adjusted**
Predictions from Model[2]	**Estimate**	**95% CI**	**Estimate**	**Estimate**	**95% CI**	**Estimate**	**Estimate**	**95% CI**	**Estimate**
Prediction Probability Point	≥ 0.55			≥ 0.45			≥ 0.40		
LOS ≥ 14 days, correct	14		11	15		14	15		13
LOS ≥ 14 days, incorrect	1		4	1		2	2		3
LOS < 14 days, correct	42		39	42		41	41		40
LOS < 14 days, incorrect	1		4	0		1	0		2
Accuracy, % correct of total	96.6		86.2	98.3		94.8	96.6		91.4
Sensitivity, %	93.3	(68.1;99.8)	73.3	100.0	(78.2;100.0)	93.3	100.0	(78.2;100.0)	86.7
Specificity, %	97.7	(87.7;99.9)	90.7	97.7	(87.7;99.9)	95.3	95.3	(84.2;99.4)	93.0
Positive predictive value, %	93.3	(68.1;99.8)	73.3	93.8	(69.8;99.8)	87.5	88.2	(63.6;98.5)	81.3
Negative predictive value, %	97.7	(87.7;99.9)	90.7	100.0	(91.6;100.00)	97.6	100.0	(91.3;100.0)	95.2

[1] maximum likelihood estimates adjusted for small sample size using Firth's penalized likelihood approach

[2] based on using prediction probability point specified for each model

Model 1 includes signature miRNAs

Model 2 includes signature miRNAs and gestational age

Model 3 includes signature miRNAs, gestational age, and the type maternal MOUD

AUC, area under receiver operating characteristic curve; CI, confidence interval; MOUD, medication for opioid use disorder; SE, standard error

Supplementary Figure S5: Pathway Enrichment Analysis of Model miRNAs

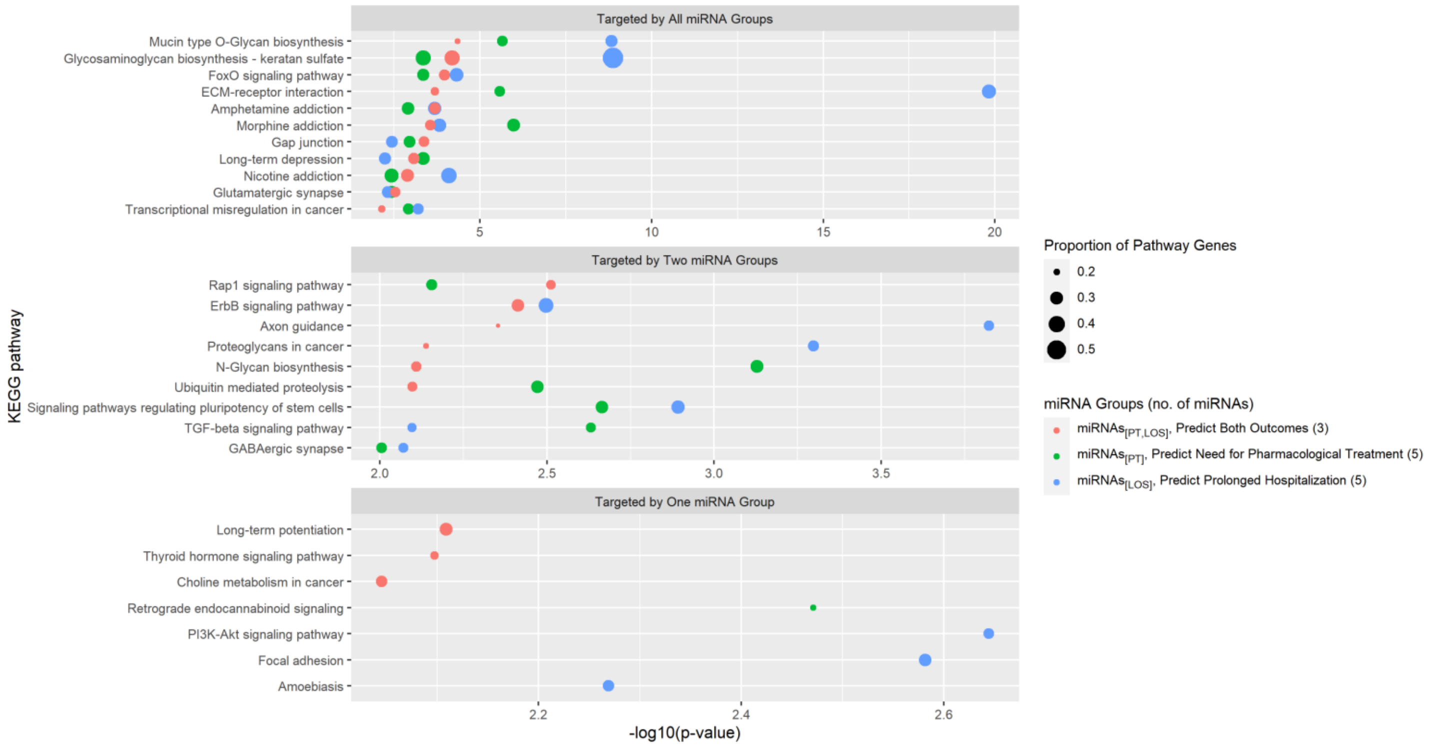

Supplementary Figure S5: Using DIANA-mirPath v3.0, we investigated the KEGG pathways [39,40] targeted by three miRNA groups: miRNAs$_{[PT,LOS]}$, the three core miRNAs that predict both outcomes; miRNAs$_{[PT],}$the five miRNAs that predict need for pharmacological treatment; and miRNAs$_{[LOS]}$, the five miRNAs that predict prolonged hospitalization. Size of points denotes the proportion of genes within each pathway that are hypothesized to be targeted by the miRNAs in each group.
